# Balancing employee flexibility and organizational performance: implications for innovation, productivity, and company attractiveness in SMEs

**DOI:** 10.3389/fpsyg.2025.1518284

**Published:** 2025-03-14

**Authors:** Ingela Eng, Emmanuel Aboagye, Eva L. Bergsten, Annika Strömberg

**Affiliations:** ^1^Faculty of Health and Occupational Studies, Department of Occupational Health, Psychology and Sports Sciences, University of Gävle, Gävle, Sweden; ^2^Department of Psychology, Norwegian University of Science and technology, Trondheim, Norway; ^3^Faculty of Education and Business Studies, Department of Business and Economic Studies University of Gävle, Gävle, Sweden

**Keywords:** small and medium sized companies, flexible work arrangements, innovation, productivity, organizational attractiveness

## Abstract

**Introduction:**

Flexible Work Arrangements (FWA) have become increasingly prevalent, offering employees flexibility in time, tasks, and location. While extensively researched at the employee level, FWAs organizational impact, particularly on innovation, productivity, and perceived organizational attractiveness, is less explored, especially in small and medium-sized enterprises (SMEs). This study aims to explore how FWAs influence these key areas from the perspective of SME managers.

**Method:**

A qualitative study used semi-structured interviews with 17 managers from SMEs in Sweden. Participants were selected based on diversity in industry, gender, and experience. Data were analyzed using thematic analysis to identify recurring themes regarding innovation, productivity, and company attractiveness in relation to FWA.

**Results:**

The results revealed that while FWAs can enhance innovation by promoting employee autonomy and diverse work settings, they may also hinder long-term innovation by reducing spontaneous discussions and collaboration. FWAs may improve individual performance but pose challenges to productivity in coordinating teams and maintaining project timelines, necessitating increased managerial oversight. Additionally, while offering an FWA attracts employees, it may weaken employee loyalty.

**Discussion/conclusion:**

These findings highlight the need for SMEs to carefully balance the benefits of FWAs with potential drawbacks, to maintain a competitive edge, suggesting the importance of structured management processes, regulated onsite workdays, and strategies to strengthen organizational connections. This balance is essential for sustaining innovation, productivity, and organizational cohesion. The findings also underscore the complexity of implementing FWAs in a way that supports long-term organizational success.

## 1 Introduction

Flexible work arrangements (FWA) are a common feature of today's work life. In some industries, it has long been a natural way of working, while in others its use has escalated in recent years (Gilson et al., 2022). FWA is commonly defined as a formal or informal agreement between employer and employee (Eurofond, 2020). Employees can adjust their working hours, for example, by being able to choose when work starts and ends (flexibility in time), how to perform work (flexibility in which tasks are carried out and in which order), and/or they can be allowed to occasionally or regularly work remotely (flexibility in space) to a variety of degrees (Eurofond, 2020; Jeffrey Hill et al., 2008). In Sweden, although the COVID-19 pandemic accelerated the transition and increase in flexible work, around 40–56 percent of the Swedish workforce already had influence over their working hours or the option to work remotely before the pandemic (European Commission, 2020). As such, the use o FWA is expected to increase in the future due to sustainability issues, including social, ecological, and economic factors like reductions in commuting time, housing demand, maintenance costs, and electricity consumption (Lundqvist and Wallo, 2023).

In general, FWA combines two perspectives, the employee's (Kelliher and Anderson, 2010) and the organization's (Reeve et al., 2012; Wattis et al., 2013). Although an array of studies has focused on FWA outcomes at the employee level, few studies have focused on the organizational level, especially in SMEs. At the employee level, FWA is associated with positive factors such as lower work-related stress (Wang et al., 2011), improved work attitudes (Kim and Wiggins, 2011; Chen and Fulmer, 2018), higher job satisfaction (Delanoeije et al., 2019; Wang et al., 2021; Hill et al., 2010), increased commitment to work (e.g., Bjärntoft et al., 2020, reduced absenteeism Hill et al., 2010, and better work-life balance Eng et al., 2024; Messersmith et al., 2011; Eurofond, 2020). Studies within large companies indicate that these positive effects can improve organizational aspects such as productivity, financial performance, and competitiveness (Bessant et al., 2005). However, the positive effects of FWAs on SMEs specifically have not been studied as extensively.

Previous research has suggested that innovation in SMEs is associated with financial performance and competitive advantage (Hassi et al., 2022; Shanker et al., 2017; Damanpour et al., 2009). Innovation is commonly posed as the process of developing new ideas, methods, products, or services that create value or improve existing solutions within companies (Zahra and George, 2002). A critical point for companies is often how they absorb innovative ideas, that is, the organization's ability to capture and transform innovative ideas into operational activities (Zou et al., 2018; James, 2014). Researchers have suggested that FWAs promote the creation of new ideas, which is favorable for the early stages of innovation (Singh and Greenhaus, 2004; Abdel Aziz and Rizkallah, 2015). As such, FWA through improved work-life balance has been suggested to act as a catalyst for innovation, where companies investing in the well-being of their employees create favorable conditions for continuous learning and creativity (Singh and Greenhaus, 2004). However, ideas at the employee level only represent the start or first phase of innovation, as innovation is a process that activates different parts of the organization (Birkinshaw et al., 2008) and cannot occur without interaction between employees or between employees and management (Shanker et al., 2017; Gibbs et al., 2024). Moreover, recent studies have found that remote and hybrid work models reduce the quality and frequency of innovative ideas, highlighting the challenges companies face in maintaining innovation without regular in-person interactions (Dwivedi et al., 2021).

The need for innovation has increased globally over the years, which has led companies to strive to recruit talent who can work innovatively within limited cost and time (Srivastava and Bhatnagar, 2008; McCrate, 2005). This indicates that an FWA as an arrangement that is attractive to employees can be used strategically to attract employees with relevant and in-demand skills, as a strategy to increase the company's competitiveness (Pepple, 2017; Stavrou and Kilaniotis, 2010).

Employees with flexibility in time or work location can experience a greater sense of control over their workday, which can have a positive impact on employee productivity, especially when managed well and tailored to the needs of both the organization and the employees. Previous research indicates that FWAs enhance employee productivity by increasing motivation (Bessant et al., 2005; Kossek et al., 2023) and engagement in work-related tasks (Kelliher and Anderson, 2010), reducing turnover (Chen and Fulmer, 2018), and improving wellbeing through perceived autonomy (Chung and Tijdens, 2013). In summary, FWAs can lead to a more motivated, loyal, and productive workforce, which strengthens the company's competitiveness and long-term success.

Larger companies may use FWAs to attract new employees with the right skills and retain existing employees (Kelliher and Anderson, 2010; Costa et al., 2004; Thompson and Aspinwall, 2009). Furthermore, if companies succeed in maximizing employee engagement and motivation through successful recruitment, it enhances their competitiveness in today's market (Azeem and Kotey, 2023). This indicates that FWA can be strategically used to attract employees with relevant and in-demand skills, thereby boosting the company's competitiveness (Pepple, 2017; Stavrou and Kilaniotis, 2010). Conversely, employees also represent a company's highest cost category (Kotey and Sharma, 2016). Therefore, it is crucial for managers to optimize their organization with employees with the right skills to improve company financial performance.

SMEs operate differently from larger companies (European Commission, 2015) and trail in adopting agreements for flexible work due to narrower margins and fewer employees. In addition, SMEs have limited financial resources compared to larger companies and are therefore cautious when faced with fierce market competition (European Commission, 2015). Further, the effects of FWAs on innovation, productivity, and talent attraction may have different implications in SMEs in comparison to large companies. Despite the many benefits of FWAs, there is still a lack of knowledge about the effects at the organizational level regarding innovation, productivity, and company attractiveness, especially in SMEs (Coenen and Kok, 2014). By examining FWAs at the organizational level, we can reach a deeper understanding of how innovation can be fostered and effectively implemented within SMEs, which is critical to their long-term success and competitiveness.

The aim of this study is to explore the consequences FWAs have on innovation, productivity, and company attractiveness from the perspective of SME managers. In studies on FWA, there is the need to highlight the limitations of focusing solely on the employee perspective rather than the broader, long-term benefits that an organizational view can offer. While employee benefits of FWAs are well-documented, an organizational perspective provides a more holistic view that is essential for long-term sustainable success. The study emphasizes the significance of FWA in these three key areas of organizational outcomes and enabling organizations to harness the full potential of FWA while mitigating associated risks.

## 2 Materials and methods

### 2.1 Study design

The study was conducted as a qualitative interview research design to explore SME managers' perceptions of how outcomes at both employee and organizational levels were influenced by FWAs. Managers' perspectives are crucial because they play a central role with dual responsibilities: ensuring the work environment and wellbeing of their staff and securing the long-term survival of the organization. Highlighting the dual responsibilities emphasizes their central role in understanding the short-term and long-term effects of FWAs on both employees and organizations. By focusing on SME managers who implement FWAs, we can gain insights into their experiences and perceptions about the economic and organizational sustainability of FWAs.

### 2.2 Sample and data collection

#### 2.2.1 Sample

SMEs were the specific target group of focus in this study. Thus, the criterion for choosing the sample had to fit the SME definition according to the European Commission European Commission (2015). The category SME includes companies with between 10 and 250 employees, where smaller companies consist of 10 to 49 employees and medium-size companies consist of 50 to 250 employees. The sample included 10 small and 6 medium-size private firms from various sectors, such as manufacturing industries, finance, consulting (business development consultants, development consultants, educational consultants), and telecommunications. In addition to the inclusion criteria, the study aimed for diversity based on gender, age, industry, and managerial experience, to diversify the information and include various perspectives.

To ensure a diverse yet analytically relevant sample, a purposive sampling strategy was used, selecting managers who had direct decision-making authority over FWAs in their organizations. This approach allowed us to capture a wide range of perspectives on FWAs' impact at the organizational level, ensuring variation across industries and managerial backgrounds

The data was collected from SMEs located in Gävleborg County, a region in central Sweden. Gävleborg represents a mix of urban and rural business environments, with a diverse SME landscape spanning manufacturing, finance, consulting, and telecommunications. This regional diversity enhances the transferability of our findings to other SME contexts, particularly in similar regions across Sweden and beyond.

Inclusion criteria required participants to be SME managers with direct knowledge of their organization's FWA policies, including decision-making on work hours, logistics, and workspace arrangements. To ensure comparability across cases, participants had to have at least 1 year of experience in a managerial role overseeing FWA implementation. Participants had to be at least 18 years old. None of the participants were compensated for their participation.

The sample size was influenced by pragmatic considerations such as the availability of SME managers who met the study's inclusion criteria and were willing to participate within the study timeframe. Given the resource constraints often present in qualitative research, the achieved sample of 17 was both practical and sufficient for capturing a range of perspectives

Sample size was also determined based on the principle of data saturation, where interviews were conducted until no new themes emerged (Guest et al., 2006; Eaton, 2003). Previous research suggests that thematic saturation is often achieved with 12–20 interviews in qualitative studies (Eaton, 2003; Konrad and Mangel, 2000). Given the focused nature of our study and the recurrence of key themes across the dataset, the final sample of 17 managers was deemed sufficient to capture the variation in SME managers' perspectives on FWAs.

#### 2.2.2 Data collection

The first author conducted one-on-one semi-structured interviews in a private room in the managers' workplace between January and June 2024. The interviews took between 60 and 90 min and were audio-recorded with the participants' consent. An interview guide followed to ensure that all relevant topics were covered along with their follow up questions. The interviews started with background questions about age, managerial experience, company objective goals, the workforce, decision-making processes, business performance indicators, and work organization. This was followed by broad questions about participants' attitudes toward FWAs, innovation, and finances. Each topic was approached with open questions so the participant could explain in their own way. Moreover, prompts for more explanation were given throughout the interview, as follow-up questions with a research goal in mind as well as a particular direction and focus were guided by the interviewer. The interviewer followed up with a few questions to ask the participants to clarify specific points. Those points were different for each interview, depending on the participant's previous answers.

The study was approved by the Swedish Ethical Review Authority (Ref. no. 2023-02441-01).

### 2.3 Analysis

In this study, the researchers used a thematic analysis method following the six phases suggested by Braun and Clarke (2022).

First, the audiotaped interviews were transcribed using Amberscript. In this step, the first author listened to the text as an audio file and made corrections in the text where Amberscript transcribed incorrectly. The transcribed interviews generated about 20–30 pages of single-spaced text. Moreover, the first author listened to the interviews several times to gain a deeper understanding of the content. The interviews were then read through to get an overview of the material.

In the second phase, we conducted the qualitative analysis of the data using ATLAS.ti 23.2.1. The first author began with an inductive coding process allowing themes emerge from the data rather than to be predefined. Initial codes were then systematically compared across interviews to ensure internal consistency and rigor. To develop and refine themes and subthemes, the coding process followed an iterative approach. After initial coding, codes were grouped into broader categories, which were then reviewed and reorganized to form overarching themes. These themes were continuously reassessed through a recursive process, where the first author returned to the dataset to verify that themes accurately reflected the data. Subthemes were developed when recurring patterns emerged within broader themes, providing additional nuance and depth to the findings. To enhance the credibility and dependability of the coding process, the following steps were taken: (1) A coding framework was developed iteratively, with adjustments made through ongoing discussions among the first and last author to refine and clarify coding categories. (2) To ensure credibility, a reflexive approach was adopted, where potential biases were acknowledged and discussed throughout the analysis process. (3) Themes were broadly outlined and illustrated with representative quotes to enhance transparency in data interpretation. In the third phase, with codes analyzed, different candidate themes were created. Fourth, coherent patterns across the dataset were formed, and themes were refined through active discussion by the first and last author. Fifth, all authors actively discussed and further refined and defined the themes to ensure coherence and alignment with the study's research questions. While the final phase of thematic analysis, as described by Braun and Clarke Konrad and Mangel (2000), typically involves extensive refinement and integration of themes into a broader narrative, our study focused on systematically defining key themes in relation to the research objectives. In this step, the first author conducted a detailed analysis of each theme, carefully delineating its boundaries by rereading the entire dataset. This process ensured clarity regarding what was included and excluded within each theme. Finally, in the sixth phase, the authors produced the final report of the analyzed dataset

## 3 Results

The present study included 17 participants, ages 34–56 (M = 46), 11 male and 6 female. When the managers reflected on innovation, productivity and company attractiveness and their connection to FWA, their answers were formed into different key themes in each aspect, as follows.

**Innovation**. (i) Innovation is a central process to business development; (ii) FWAs may facilitate innovative ideas yet inhibit the growth of ideas; (iii) the long-term consequences of FWA for innovation power must be managed.

**Productivity**. (i) It is a struggle to monitor productivity. (ii) FWAs create an increased need for management involvement; (iii) there must be regulation to reduce unpredictability in productivity.

**Attractiveness of the company**. (i) FWAs attract skills and competence; (ii) there are challenges with loyalty.

### 3.1 Innovation

#### 3.1.1 Innovation is a central process to business development

It was common across cases that innovation was held to be an important process for business development. Managers explained that although innovation starts with an idea, the idea must grow and be refined in a chain together with colleagues to become innovation for the company. Managers expressed that innovation comes in a variety of forms and is not limited to single groundbreaking interventions. Some variations involved discovering new methods to address work-related issues, enhancing existing practices, developing innovative solutions for tasks, generating ideas that contributed to business growth, creating new services for customers, or leveraging existing inventions for competitive advantage.

#### 3.1.2 FWAs may facilitate innovative ideas but inhibit the growth of ideas

According to the managers, FWAs that allow employee autonomy and varying work locations can facilitate innovative ideas. However, they highlighted that innovation requires collaboration and teamwork across all organizational levels. That is, effective innovation relies on synchronized efforts and coordination between employees, but work location was not crucial:

*When we work with product development… It's a huge team effort. For us, product development is a project. /…/. So it doesn't work if everyone just does what they want, it requires a pretty hard level of synchronization and that people know what to do. And my role is to see to that they are interacting and coordinated. (Male, 38, 16 years of total management experience, manufacturing industry)*.

In contrast, some managers are of the view that creative discussions among employees to refine ideas may be lost with FWAs: “*Spontaneously. The given answer is that it would decrease as it becomes more difficult with innovation. For innovation, I would say… It's best when you see each other… You should have lively discussions”. (Male, 34, 9 years of total management experience, manufacturing industry)*.

Moreover, working onsite makes it more likely to meet other employees with diverse knowledge, work experience, and backgrounds within work groups, which can be seen as beneficial to developing innovations: “*It's usually very rewarding and like if you're talking about early stages like product development… and you are a group with different personalities but also have different prior knowledge and professional backgrounds.” (Male, 44, 12 years of total management experience, manufacturing industry)*.

Another challenge lies in how employees disseminate ideas and innovations beyond those who have the relevant experiences:

*But often our challenge is… How do we spread it outside to a person who has those experiences? We can have a lot of skills transfer… And it is an embryo of innovation… But it is, I think, extremely difficult to get it to stick. /…/ No matter how good and brilliant the idea is, I don't have the experience or have been involved or something to connect it to. (Male, 49, 20 years of total management experience, consulting business)*.

#### 3.1.3 Managing long-term consequences of FWAs for innovation power

The managers are also uncertain about FWAs and the long-term consequences of their use regarding innovation. Some expressed a concern that ideas never progress to the next level if they are not shared within the organization (for example, with another employee, a team, or managers). This may lead to a potential weakening of companies' innovative power over time*: “I think it can be good for the individuals periodically, but I think for the company in the long term…. If you just have distance all the time and without any rules, I don't think it's good for the company's innovation.” (Female, 54 16 years of total management experience, consulting business)*.

Managers believe that while FWAs can be beneficial for employees, their use poses a risk for organizational innovation if flexible work practices are managed poorly. Therefore, managers must balance employees' needs for FWAs with the need to maintain innovative power. This requires more disciplined leadership and structured work processes to facilitate innovation in organizations with FWA:

*Then I've tried… Distance innovation and then in different formats and workshops also at distance. And that's fine too. There are also many good tools and… But it requires more of leadership, more of discipline and also to make innovation happen during those circumstances. It does not happen as naturally, it needs to be controlled much more. (Male, 38, 16 years of total management experience, manufacturing industry)*.

### 3.2 Productivity

#### 3.2.1 Struggle in monitoring productivity

Managers expressed the view that FWAs require clear deliverables to track productivity against business goals. Although employee productivity might increase, managers struggle to monitor the overall productivity and calculating delays caused by such agreements:

*The perceived productivity of the person who work remotely is like… increases significantly then. It's a well-known fact, I guess. ‘Now that I work here, it works super well. No one bothers me.‘ No, fine. That can be the case with quite a few tasks for a limited time anyway. That way I really got work done well. But these three people who want to get hold of me… got a delay. Maybe because It wasn't available. I didn't sit next door to them and then I lowered their productivity instead by being away. This is very difficult to analyze the effects of. (Male, 44, 12 years of total management experience, manufacturing industry)*.

When a project needs to be solved collaboratively in groups or teams, difficulties controlling the project can arise. The managers experienced problems with management and control of projects when employees are working remotely and not synchronously. That situation can also lead to communication problems among the employees within the project, which slows down the overall project time. In some companies, they have already decided to implement certain restrictions regarding remote work:

*Yes, it in our project that we experience inertia. What is the inertia due to? /…/ Now we have decided to back off a bit on flexibility. So they [corporate management] have realize that it has to do with this [flexibility]. But we can't prove it. (Male, 54, 14 years of total management experience, manufacturing industry)*.

Although managers expressed that FWAs may have the potential to inhibit group productivity due to the need for collaboration and coordination and yet for tailor-made flexible settings for the individual employee, they highlighted the importance of having clear goals or project goals. Collaboration and communication are keys to reaching these goals: *And with a development project… Then we are talking about maybe 4000 people. In a team effort and interact and sync to reach a final goal. In that case everyone can't do what they want. (Male, 34, 1,5 years of total management experience, manufacturing industry)*.

Another manager highlighted that feeling part of a team with colleagues working toward the same objectives is vital: “*Whatever we want to call it, you feel like you're here [at the main workplace] and you have colleagues who… do the same things and want to go in the same direction.” (Male, 38, 16 years of total management experience, manufacturing industry)*.

#### 3.2.2 Increased need for management involvement

According to the managers, FWAs demand more management involvement in distribution of tasks between employees and in balancing workloads. Managers found it easier to manage and coordinate tasks when employees worked onsite, and they experienced greater responsibility to facilitate workflow for beneficial productivity when there were FWAs. Therefore, managers' report that they spend more time on individual check-in meetings and task distribution to maintain workflow efficiency:

*But with support from me if you need it. Or priorities if it becomes too much. Then I have to step in and say but okay, 'now we'll put this away.' Or ‘don't give a damn about this now‘. Or, I also have to be clear when I… Or always try to be clear when I hand over a task to ‘please note, this is when you have time‘ or ‘this is a long task that you can work on for 6 months now‘. (Female, 54, 16 years of total management experience, consulting business)*.

The managers experience a greater need to have an overview of work processes and employee workload in order to balance the tasks for employees working with FWAs. They highlighted a risk of employees working “in the wrong direction” without having the path pointed out:

*If you work a lot alone, you may go in one direction for a very long time. Until someone else pokes you a little bit at it. What if it will be too late then? Then it might be wasting a lot of time there and it could have been more efficient if you and the groups had been allowed to work together on this and sort of find all the angles from the beginning and then, ‘Okay now, now we start running more in this direction'. (Male, 44, 14 years of total management experience, manufacturing industry)*.

Some managers described employees being unsure of how to solve certain tasks and handle different situations as normal. Working onsite, help is perceived as more available, whereas FWAs create an invisible barrier between employees and with managers. Consequently, managers are concerned that FWAs have the potential to affect overall productivity negatively because employees get stuck on tasks, causing uncertainty and delays. They expressed that even though FWAs promote employee balance, employees might solve work tasks incorrectly without managers or other employees noticing, leading to the need to do work over again:

*I feel that we still have a lot of uncertainty linked to the fact that people may not always know what to do in all situations and that creates that… creates uncertainty and things take longer than it would need. (Male, 54 14 years of total management experience, manufacturing industry)*.

#### 3.2.3 Regulation to reduce unpredictability in productivity

Some managers described that in the sense of productivity and the company's best interest, FWAs needed to be regulated to optimize flexibility and productivity. Managers suggest predetermined onsite workdays could reduce project delays and inefficiency caused by unsynchronized FWAs:

*As it is now, it [FWA] contributes to reduced sync and thus reduced efficiency. And then it clearly leads to that frustration that I talked about that many people believe. I get frustrated by this unpredictability in delivery and progress. It is a negative effect. Otherwise, it's purely psychosocial. I think so. One thing that I have heard we are discussing or have been discussing for some time is whether we should decide one or a few days a week when you are on site. So we don't deviate from this 3 days a week rule or 50%, but that we … that you decide that Tuesday and Thursday then we are at the office and then you get to choose a day free [where work is located] or whatever it could be…. So that's probably another negative aspect of the fact that we have full freedom basically like today. (Male, 38, 16 years of total management experience, manufacturing industry).*.

Some companies have already chosen to step back from unregulated remote work:

*But I will say that based on that, the company sets and now the management company has also decided to back down. They will demand it. Everyone has to be on site 3 days a week because we have seen that there is a connection between the projects and the progress over the fact that it is slow. Oh well, completely mad. (Male, 54, 14 years of total management experience, manufacturing industry)*.

### 3.3 Attractiveness

#### 3.3.1 Attracting skills and competence

Managers highlighted the importance of employees with the right competences in the organization as a strategy to reach the company's goals. Managers believed that FWAs may attract the right talent both by retaining current employees and attracting new employees with required skills. This belief was confirmed to some extent by an increase in application for the positions, a larger geographical recruitment area, leading to a larger selection of candidates where they could screen to find the best suited employees in competition with other companies:

*But for us, it has been a huge strength, I would say. Because what we achieve by being flexible in this way is competence that we would otherwise never have. Something that has been completely critical for what we are trying to do. So that's probably also an aspect of it. (Male, 38 2 years of total management experience, information technology and business services sector)*.

And another manager highlighted that the purpose of FWAs is to attract employees with the right skills: “*Yes, it is… I would say that this is the purpose of attracting the right people…. But there is a very interesting message in the matter of deciding for yourself, governing yourself but still taking responsibility for.” (Male, 49, 20 years of total management experience, consulting business)*.

#### 3.3.2 Challenges with loyalty

Managers are concerned about the challenge of maintaining employee loyalty with FWAs especially when employees work remotely. They emphasized the importance of shared vision and values, which is easier to cultivate when working onsite. FWA was seen as creating weaker ties within the organization and increasing the risk of employees being headhunted by other companies:

*I want to talk about this thing about actually attracting and continuing to attract as well…. And then we talk about this negative or positive thing that may not be reinforced in the same way when you don't see each other in everyday life. Then I think, if I talk at the same time as I think, in the long run, if you work too much remotely, the question is whether you will be as loyal or equal… Maybe it could make the step to changing employers much easier so that you don't get this one. Maybe the same social feeling, connection, belonging. (Female, 48, 20 years of total management experience, financing)*.

The connection between employees and the company can become very weak with remote work. Being in the office, meeting colleagues, and building bonds creates a sense of coherence, making it easier to retain staff. This connection together with enjoyment of the workplace make it harder for employees to leave, even despite numerous job offers:

*What are we as employers? … The connection between me and (the company name) may be very, very weak. I think. It can be… If I'm in the office and meet colleagues and tie those ties and kind of feel like I have a context and a coherence. Then it may also be easier to retain staff. Or very straight… But I think it's harder for someone to change a job if you have that kind of connection and enjoy the company and feel that community than if you sit like I could do here in [work location at a distance] for a year and just be away from there…. I get ten work-proposals a week 'Come to us, come to us. We have this, this, this, this, this, this'. But I still have the connection and my colleagues up there (the company's main location) that I like and kind of believe well in the company. But is that what has made me stay? Presumably. Otherwise, I have 100 opportunities to make a career. So I think… to come as a brand new one and just sit remotely, then you will never have that connection to why should I be at this particular company? What is it even besides the one that gives me a salary? Sort of. (Male, 38, 2 years of total management experience, information technology and business services sector)*.

## 4 Conclusion, discussion, future implications, and limitations

### 4.1 Conclusion and discussion

Previous research has often explored themes of innovation, productivity, and company attractiveness in the context of FWA, but few studies have focused specifically on their impact within SMEs. This study provides a nuanced understanding of how FWAs influence these areas from the perspective of SME managers. The findings reveal that while FWAs offer significant benefits in terms of innovation, productivity, and attracting and retaining talent, they also introduce challenges that require careful management.

#### 4.1.1 Innovation

Studies on FWAs often portray them as a catalyst for innovation by creating favorable conditions for employee creativity and attracting innovative talent (e.g., James, 2014; McCrate, 2005; Pepple, 2017). One key finding revealed that FWAs can both stimulate and hinder innovation in SMEs. On the one hand, flexible work, through employee autonomy and diverse work locations, can spark innovative ideas. On the other hand, onsite work facilitates spontaneous discussions and knowledge sharing, which are vital for innovation. Furthermore, effective innovation often depends on teamwork and synchronized efforts, which remote work can compromise.

Therefore, there is concern about the long-term consequences of FWAs on innovation, as ideas may not progress if not shared within the organization, potentially weakening innovative power over time. Our results can be interpreted as an indication that FWAs have advantages and disadvantages when it comes to innovation in SMEs. To navigate the complexities of both positive and negative consequences, management and structured work processes are required to balance flexible work with the need for collaboration and interaction. Managers need to find ways to promote both autonomy and teamwork to ensure that innovation can thrive in an environment with FWAs. Active management and coordination are crucial to maintaining innovative capacity and mitigating their potential negative effects.

#### 4.1.2 Productivity

In terms of productivity, the key findings were that monitoring employee productivity in a company with FWAs can be challenging. On the one hand, and in line with previous studies, employee productivity might increase (Bessant et al., 2005; Kossek et al., 2023; Bloomberg, 2007; Konrad and Mangel, 2000). On the other hand, the overall productivity and project timelines can suffer due to coordination and communication issues. Managers have rediscovered that they need to be more involved in task distribution and balancing workloads, spending more time on check-ins and task management to maintain workflow efficiency and prevent misdirection. These check-ins and task management are not to be confused with control by managers; rather, they are about ensuring company results and delivery. Managers in our study suggested that regulated onsite workdays can optimize flexibility and productivity by reducing project delays and inefficiencies caused by unsynchronized FWAs.

Our results highlight specific challenges for SMEs, such as difficulties in coordinating teams and projects and the need for increased management involvement. One interpretation of our results could be that FWAs create challenges in monitoring and maintaining productivity. Management and regulated FWA practices are necessary to balance the benefits of these agreements and ensure that productivity is not negatively affected. Managers must adapt their involvement and work processes to address the unique challenges posed by FWAs.

#### 4.1.3 Attractiveness

Another key finding concerning the consequences of FWAs was that they facilitated attraction and retention by offering flexibility, leading to an increase in job applications and a broader geographical recruitment area. However, maintaining employee loyalty is still challenging in the case of FWAs. Remote work can weaken ties to the organization, making it easier for employees to be recruited by other companies. Onsite work helps cultivate a shared vision and values, fostering a stronger connection to the organization.

While previous studies emphasize the strategic advantages of FWAs in SMEs (Kotey and Sharma, 2016), our results provide a more nuanced picture by incorporating specific experiences and challenges, particularly concerning loyalty and social ties within the company. Although FWAs can attract and retain talent, they may also apparently weaken employee loyalty to SMEs, underlining the need for strategies to maintain strong organizational connections.

### 4.2 Implications for Organization

Our study highlights the complexity of balancing innovation, productivity, and company attractiveness in SMEs that incorporate flexible work. Our study shows that there are challenges in the use of FWAs. What appears positive for the employee is not always positive from an organizational perspective. This is important for SMEs to keep in mind to avoid losing their organizational perspective and to guide how they should work with FWAs in their organization.

#### 4.2.1 FWA policies

A practical contribution of our study is that it provides a basis for internal discussions within companies to achieve a common view on the opportunities and challenges of FWAs. This can then serve as a foundation for creating policies and guidelines for those who feel they need them. Clear policies to create would include which types of flexible work methods are allowed (for example, remote work, flex time) expectations for availability, and technical requirements. Then guidelines could be established and communicated around FWAs in organizations. Future organizations should explore, develop, and adapt strategies, tools, and methods for flexible work agreements that benefit both employees and the organization to achieve company goals and strengthen competitiveness. Continuous feedback should regularly be gathered from employees and managers about how the FWA process is working and what improvements can be made. Organizations need to adapt FWA policies based on feedback and changing business needs and continue to optimize them to ensure they support both employee and organizational goals.

#### 4.2.2 Innovation-promoting culture

To promote long-term innovation, organizations can foster an innovation-promoting culture where innovation is valued and supported, even when employees are working flexibly. This space for creativity can be created by enabling spontaneous idea generation and exchange or virtual brainstorming sessions and digital collaboration platforms. Additionally, exploring which types of collaborative digital tools and leadership methods best support innovation when employees are geographically dispersed and work at different times is essential. Furthermore, future organizations can explore how to encourage and stimulate meetings and exchanges of ideas on digital platforms to capture and develop innovative ideas.

#### 4.2.3 Clear goals and expectations for productivity

To maintain or enhance productivity, future research could investigate methods and tools for following up on employee productivity in remote or hybrid work environments. Organizations can work in a goal-oriented way by shifting the focus from surveilling working hours to measuring performance and results. Managers can set clear goals and expectations and evaluate employee performance based on achieved results that contribute to the organization's goals rather than hours worked. Moreover, future studies should examine how various leadership styles and methods must be adapted to maintain or promote productivity and coordination in FWA environments. Additionally, it is important to explore different structures' pros and cons and different policies for FWAs in different industries. These structures include predetermined onsite workdays and their consequences on productivity.

#### 4.2.4 Strategies for a sense of belonging

For company attractiveness and loyalty, future studies could focus on investigating which forms of FWA affect talent attraction and retention, and which factors promote loyalty and engagement among employees. Finally, research could explore how leadership can convey and strengthen corporate culture in FWA environments, as well as examine strategies to enhance employees' sense of belonging and engagement to ensure retention.

#### 4.2.5 Structured approaches to enhance collaboration and innovation

To further mitigate the potential negative consequences of FWAs on collaboration and innovation, SMEs could adopt a hybrid work model that combines remote work with regular, scheduled in-person sessions. These sessions can help maintain spontaneous idea-sharing and strengthen teamwork. In addition, implementing structured digital communication protocols (such as regular virtual brainstorming sessions and scheduled team check-ins) can facilitate continuous collaboration. These measures offer actionable strategies for SMEs to balance flexibility with the need for organizational cohesion and sustained innovation.

### 4.3 Limitations and suggestions for future research

This study highlights the consequences of FWAs on innovation, productivity, and company attractiveness in SMEs, but there are limitations that suggest opportunities for future research. The relatively small sample limited to one county in Sweden may affect the generalizability of our findings. Future research should consider a larger and more geographically diverse sample of SMEs to enhance external validity and improve generalizability. The sample included a selection of industries, but the nature of work plays a role in how FWAs are implemented and experienced. Future studies can examine more industries to enrich the diversity of FWA experiences. Given that this study used a qualitative method through interviews, no causal effects are inferred. Future studies could use a mixed methods or longitudinal design to better explain the direct association on the long-term impacts of FWA on innovation, productivity, and attractiveness in SMEs.

## 5 Conclusion

Our study shows that it is important not only to consider flexible work agreements from an employee perspective but also the more long-term consequences at the organizational level. These two perspectives need to be balanced in order to achieve innovation, productivity, and attractiveness for a long-term sustainable organization. Our results indicate that while FWAs can boost innovation by promoting employee autonomy and diverse work settings, they may also impede innovation due to the reduction in spontaneous discussions and the knowledge sharing that occurs in onsite work. Regarding productivity, employee performance might improve with FWAs, but overall project timelines could suffer from coordination challenges, necessitating more managerial oversight. Additionally, FWAs can attract and retain talent by providing flexibility and expanding recruitment reach, yet it may diminish employee loyalty. Management, structured processes, and regulated onsite days are crucial for balancing the advantages and drawbacks of FWAs, ensuring ongoing innovation, productivity, and employee commitment in SMEs. Understanding and addressing these consequences of FWAs on innovation, productivity, and attractiveness are crucial for the long-term sustainability and competitiveness of organizations, particularly SMEs.

## Data Availability

The raw data supporting the conclusions of this article will be made available by the authors, without undue reservation.
